# Serological assessment of SARS-CoV-2 infection during the first wave of the pandemic in Louisville Kentucky

**DOI:** 10.1038/s41598-021-97423-z

**Published:** 2021-09-14

**Authors:** Krystal T. Hamorsky, Adrienne M. Bushau-Sprinkle, Kathleen Kitterman, Julia M. Corman, Jennifer DeMarco, Rachel J. Keith, Aruni Bhatnagar, Joshua L. Fuqua, Amanda Lasnik, Joongho Joh, Donghoon Chung, Jon Klein, Joseph Flynn, Marti Gardner, Shirish Barve, Smita S. Ghare, Kenneth E. Palmer

**Affiliations:** 1 James Graham Brown Cancer Center, University of Louisville School of Medicine, University of Louisville, Louisville, KY USA; 2 Center for Predictive Medicine for Biodefense and Emerging Infectious Diseases, University of Louisville School of Medicine, University of Louisville, Louisville, KY USA; 3 Department of Medicine, University of Louisville School of Medicine, University of Louisville, Louisville, KY USA; 4 Department of Pharmacology and Toxicology, University of Louisville School of Medicine, University of Louisville, Louisville, KY USA; 5 Department of Microbiology and Immunology, University of Louisville School of Medicine, University of Louisville, Louisville, KY USA; 6 Christine Lee Brown Envirome Institute, University of Louisville School of Medicine, University of Louisville, Louisville, KY USA; 7 Diabetes and Obesity Center, University of Louisville School of Medicine, University of Louisville, Louisville, KY USA; 8 Alcohol Research Center, University of Louisville School of Medicine, University of Louisville, Louisville, KY USA; 9 Norton Cancer Institute, Norton Healthcare, Louisville, KY USA

**Keywords:** Biotechnology, Diseases, Infectious diseases, Infectious diseases, Immunology, Adaptive immunity, Humoral immunity

## Abstract

Serological assays intended for diagnosis, sero-epidemiologic assessment, and measurement of protective antibody titers upon infection or vaccination are essential for managing the SARS-CoV-2 pandemic. Serological assays measuring the antibody responses against SARS-CoV-2 antigens are readily available. However, some lack appropriate characteristics to accurately measure SARS-CoV-2 antibodies titers and neutralization. We developed an Enzyme-linked Immunosorbent Assay (ELISA) methods for measuring IgG, IgA, and IgM responses to SARS-CoV-2, Spike (S), receptor binding domain (RBD), and nucleocapsid (N) proteins. Performance characteristics of sensitivity and specificity have been defined. ELISA results show positive correlation with microneutralization and Plaque Reduction Neutralization assays with infectious SARS-CoV-2. Our ELISA was used to screen healthcare workers in Louisville, KY during the first wave of the local pandemic in the months of May and July 2020. We found a seropositive rate of approximately 1.4% and 2.3%, respectively. Our analyses demonstrate a broad immune response among individuals and suggest some non-RBD specific S IgG and IgA antibodies neutralize SARS-CoV-2.

## Introduction

Severe acute respiratory syndrome coronavirus 2 (SARS-CoV-2) has spread rapidly leading to a worldwide pandemic with more than 176 million infections and nearly 4 million deaths worldwide (as of June 16, 2021). SARS-CoV-2 infection consists of a broad spectrum of symptoms ranging from asymptomatic to severe COVID-19, a disease characterized by symptoms such as: fever, severe cough, anosmia, gastrointestinal symptoms, hypercoagulability, inflammatory complications, acute respiratory distress syndrome, and death^1–4^. Currently, evidence is lacking on the physiological basis for the broad range of symptoms, though individual’s immune response is thought to play an important role. Additionally, many believe that the limited pre-existing immunity to this virus has resulted in the rapid spread around the world. Historically, the true prevalence of infections exceeds the detected cases in respiratory viral epidemics^3,5^, which means individuals may not know they were infected with SARS-CoV-2 and communities may underestimate prevalence. Evidence suggests that prior infection provides immunity (with unknown duration) but there remain important knowledge gaps regarding spread and herd immunity against SARS-CoV-2. Licensed vaccines against SARS-CoV-2 have only very recently become available leading policymakers to grapple with the most effective and equitable distribution of vaccines. There is broad agreement that frontline healthcare workers should be prioritized for vaccination, but given a rapidly increasing incidence of infection, there is a reasonable case to be made for using serological markers of historical infection as one of the ways to prioritize vaccination of the immunologically naïve^5–7^. Therefore, high quality serology assays are urgently needed to aid understanding of the spread of infection and immunity, as well as the development and deployment of vaccines against SARS-CoV-2.

The SARS-CoV-2 genome encodes four structural proteins: the envelope protein, the membrane protein, the nucleocapsid (N) protein and the membrane glycoprotein known as spike (S)^8,9^. N protein enters host cells with viral RNA and facilitates virus replication along with virus particle assembly and release^10,11^. N protein has previously been shown to be highly immunogenic and has been used in vaccine development^11–13^. S directly mediates binding to host cells, specifically through its interaction with the human receptor angiotensin converting enzyme 2 (ACE2)^9^. The S1 region of S protein contains the receptor binding domain (RBD)^8^, the target of many neutralizing antibodies^9^. Neutralizing antibodies play an important role as an immune product for protection and treatment against viral diseases^2^. Levels of neutralizing antibodies have been previously used as a standard to evaluate the efficacy of vaccines and in the utilization of plasma infusion as a passive antibody therapy^2^. Serum neutralization can be measured by the gold standard plaque reduction neutralization assay (PRNT) and/or a higher throughput microneutralization assay. The use of enzyme-linked immunosorbent assay (ELISA) with recombinant antigen substrate is simple, does not require the use of a biosafety level 3 (BSL-3) laboratory, and can correlate with neutralization. Therefore, ELISA can be used as a facile screening tool for the presence of antibodies with potential to neutralize infection.

Currently, ELISAs are being developed to detect and measure the presence of antibodies against SARS-CoV-2. Recent studies have evaluated varied combinations of IgG, IgA, and IgM antibodies against S, N, and RBD in patient serum^5,9,14–17^. However, there is a need to continuously evaluate IgG, IgA, and IgM antibodies against S, N, and RBD within different patient populations to provide a better understanding of COVID-19 as well as to assess the duration and the extent of antibody responses to natural infection. Here we report the development and qualification of ELISA-based assays that analyze the presence of IgG, IgM, and IgA antibodies against the S protein, RBD, and N antigens of the SARS-CoV-2 virus. Utilizing the S and RBD ELISAs, we evaluated correlations with SARS-CoV-2 neutralization. These assays were used to analyzed approximately 2300 healthcare workers from University of Louisville Hospitals in Louisville, Kentucky during two separate months during the first wave of the local COVID-19 pandemic, May and July 2020. The goals of this study were to: (1) to perform ELISA for antibodies against SARS-CoV-2; (2) understand how primary virus neutralization correlates with the microneutralization and ELISA analysis; (3) determine seroconversion of local healthcare workers and longevity of immune responses; and (4)identify healthcare workers who may be useful donors of convalescent sera.

## Results

### Method validation

**Figure 1:**
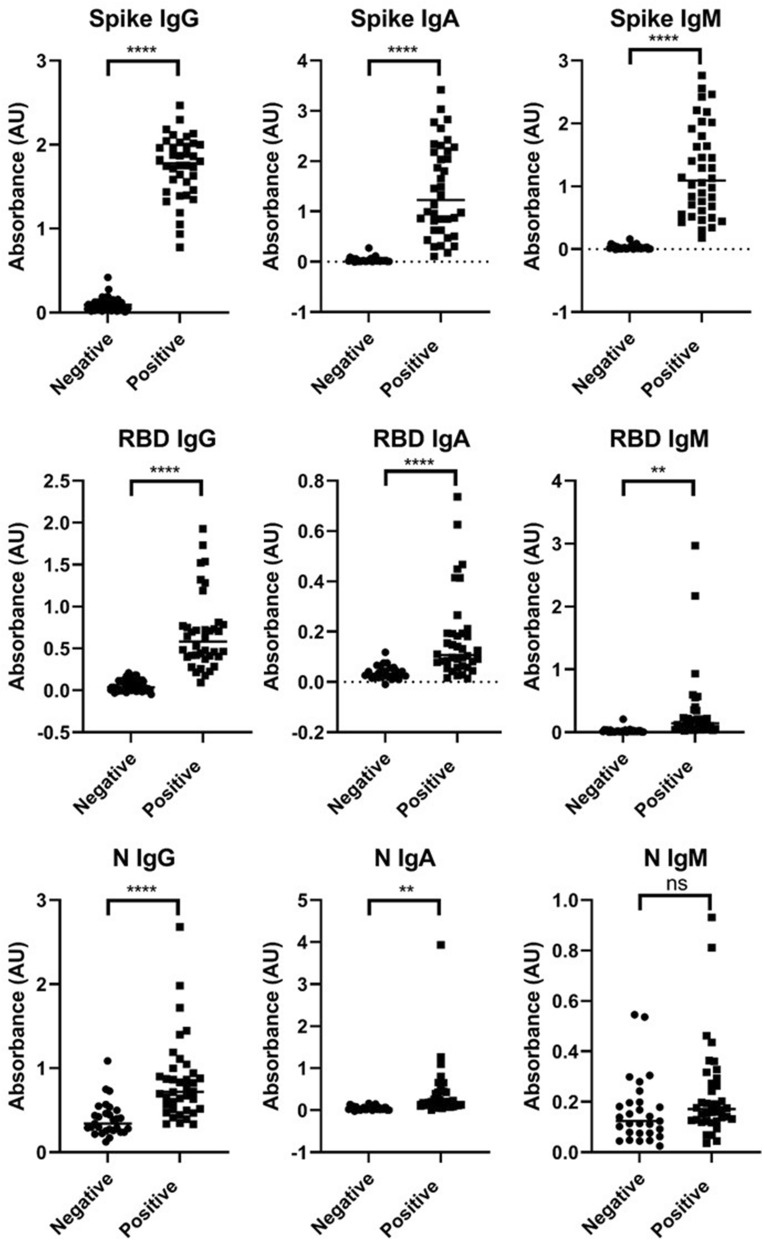
ELISA analysis of true positive and true negative patient samples. Patient sera was ran at a 1:100 dilution, n = 2. The average absorbance values are plotted. *P < 0.05, two tailed t-test (GraphPad Prism 8.0).

**Figure 2:**
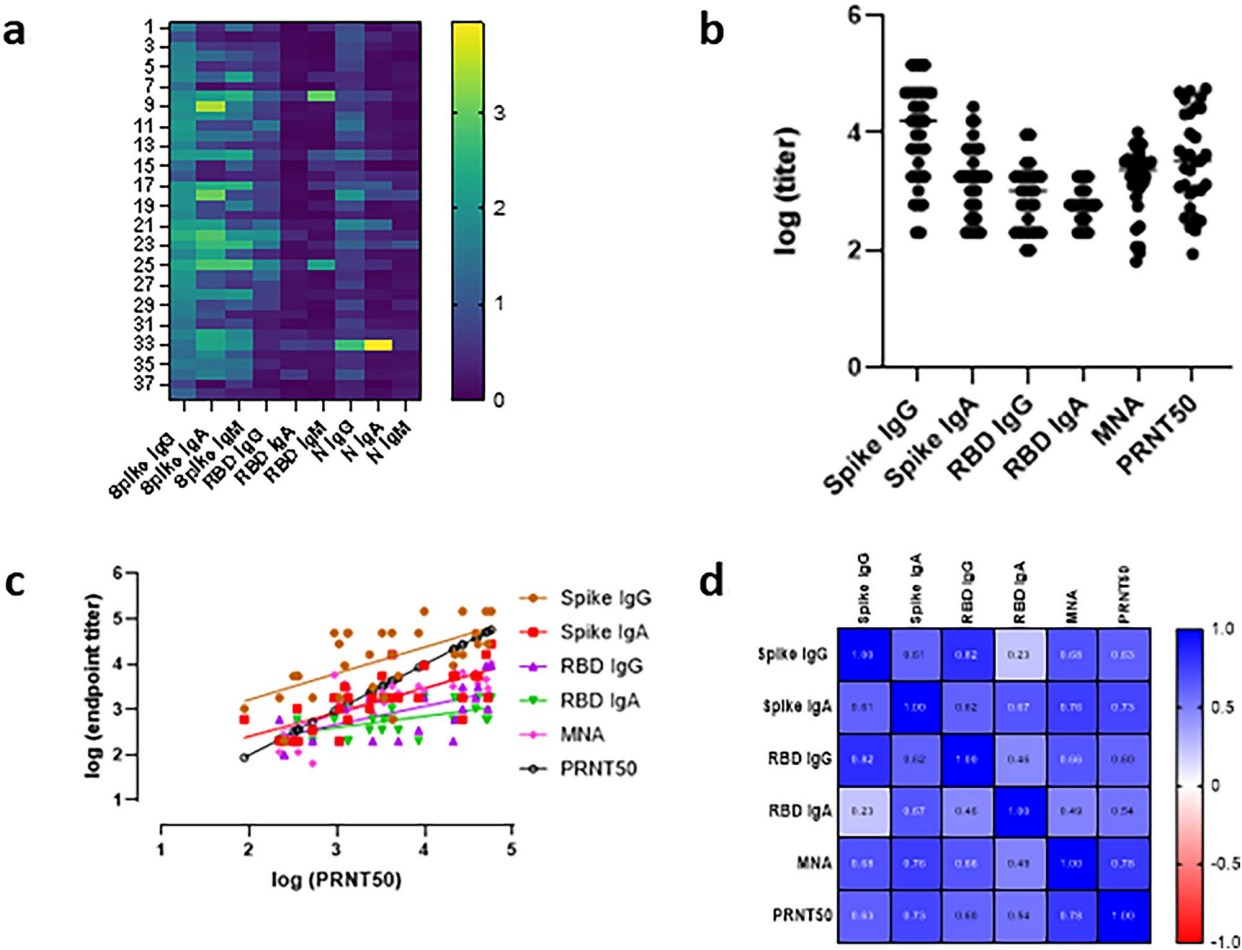
Analysis of 38 SARS-CoV-2 positive patients. (**a**) Heatmap of ELISA OD values. Samples ran at a 1:100 dilution. (**b**) Antibody endpoint titers by ELISA (n = 2), microneutralization (n = 6) and PRNT_50_ (n = 2). Antigen coated on microtiter plate, block, add serial dilution of serum, detect with labelled class specific secondary antibody. (**c**,**d**) Correlation (Pearson, p = 0.05) ELISA and microneutralization with PRNT_50_.

Using commercially-available, purified SARS-CoV-2 recombinant antigens, we optimized ELISAs for the detection of SARS-CoV-2 antibodies in 1:100 diluted human serum samples. ELISAs include detection of IgG, IgA, or IgM antibodies against S, RBD, and N antigens. A total of 38 SARS-CoV-2 positive and 29 SARS-CoV-2 negative patient sera were used to qualify ELISAs for IgG, IgA, or IgM to S, RBD and N proteins. The positive patient cohort was confirmed by RT-PCR for active virus and were positive for SARS-CoV-2 antibodies based on the Premier Biotech COVID-19 IgG/IgM Rapid Test Cassette. The negative cohort includes 29 patient sera that were negative for active virus (determined by RT-PCR) and SARS-CoV-2 antibodies based on the Premier Biotech COVID-19 IgG/IgM Rapid Test Cassette. ROC analysis was performed to determine the OD cutoff values providing optimal diagnostic specificity and sensitivity for each ELISA (Table 1). All ELISA methods except N IgM demonstrate a statistical difference between the positive and negative cohort (Fig. 1). Heat map analysis (Fig. 2A) of samples ran at a 1:100 dilution provides semi-quantitative picture of the wide variation of immune response in SARS-CoV-2 positive patients.

**Table 1:** Analytical performance characteristics for ELISAs.

	Spike (S)			RBD			Nucleocapsid (N)		
	IgG	IgA	IgM	IgG	IgA	IgM	IgG	IgA	IgM
Sensitivity	100	94.7	100	94.7	47.4	81.6	84.2	86.8	84.2
Specificity	96.6	100	100	100	100	96.6	76.0	86.2	48.3
Cutoff	0.418	0.297	0.177	0.216	0.125	0.084	0.465	0.101	0.117

ROC analysis to determine ELISA cutoff values (arbitrary units (AU)) with optimal sensitivity and specificity. Data for S IgA, SIgM, RBD IgA, RBD IgM, N IgG, N IgA, N IgM includes a total of 38 RT-PCR confirmed positive patient sera and 29 RT-PCR confirmed negative patient sera for SARS-CoV-2 antibodies. Data for S IgG and RBD IgG includes a total of 38 RT-PCR confirmed positive patient sera and 80 confirmed negative patient sera (RT-PCR or pre COVID).

To increase the quality of the S IgG and RBD IgG ELISAs, additional negative patients were added. In addition to the 29 negative samples mentioned above, 9 serum samples of individuals who were RT-PCR negative and 42 pre-COVID-19 sera were analyzed. The cutoff for the S IgG ELISA was determined to be 0.418 absorbance units (AU) giving a sensitivity of 100% and specificity of 98.8%. The cutoff for the RBD IgG ELISA was determined to be 0.216 AU giving a sensitivity of 95% and specificity of 100% (Table 1).

### Correlation with neutralization

Correlation of antibody titers with neutralization was determined based on a subset of patients divided into tiers based on their antibody titers. RBD IgG ELISA results from ELISA validation were used to assign tiers: negative (< 0.22 AU, low (0.22–0.50 AU), medium (0.50–1.25 AU) and high (> 1.25 AU). The sera of these 38 patients were tested by serial dilution on S IgG ELISA, S IgA, RBD IgG ELISA, RBD IgA, microneutralization assay, and PRNT assay (Table 2; Fig. 2B). Correlation of ELISA and microneutralization endpoints to PRNT were determined by the Pearson’s rank test in GraphPad Prism (Fig. 2C,D). There was a positive correlation with all antibody types and neutralization. Microneutralization and PRNT had a positive correlation r value of 0.78, and using PRNT as the comparator method, microneutralization has percent positive and percent negative agreement 97 and 83%, respectively.

**Table 2:** Antibody endpoint titers by ELISA, microneutralization and PRNT_50_.

Tier	Patient	SPIKE IgG	SPIKE IgA	RBD IgG	RBD IgA	MNA	PRNT50
High	1	145,800	9353	1800	1800	2580	9812
	2	145,800	600	3118	0	2896	26,950
	3	48,600	5400	1800	1039	6502	37,905
	4	48,600	1800	1800	600	5793	922
	5	145,800	16,200	9353	600	4598	49,767
	6	145,800	28,059	9353	1800	10,321	57,037
	7	28,059	1800	1039	600	2896	52,227
	8	48,600	1800	1800	1039	1625	4185
	9	9353	3118	1039	1039	3649	1214
	10	16,200	1039	1800	346	1825	2327
Medium	11	16,200	16,200	1800	1800	3251	4225
	12	28,059	200	1039	0	1825	1049
	13	48,600	1800	600	200	1149	1329
	14	28,059	5400	3118	1800	3649	40,380
	15	48,600	5400	1800	346	2435	3238
	16	48,600	600	600	200	2896	1314
	17	48,600	1800	346	200	3251	8476
	18	16,200	1800	1039	600	6502	26,867
	19	1800	200	600	200	114	219
Low	20	5400	1039	200	600	575	348
	21	3118	1800	200	600	813	2532
	22	5400	1800	200	346	2299	4968
	23	16,200	5400	600	1800	6502	21,970
	24	9353	5400	200	600	3251	21,015
	25	1800	1039	200	0	1290	1063
	26	1800	346	200	200	64	521
	27	200	200	0	0	0	0
	28	5400	200	200	0	228	317
Negative	29	600	346	100	600	91	73
	30	600	1800	0	600	1722	4293
	31	600	346	0	600	0	0
	32	0	0	0	0	0	0
	33	5400	200	0	0	114	357
	34	1800	1800	0	600	1444	3399
	35	1039	600	0	0	0	0
	36	0	0	0	0	0	0
	37	0	0	0	0	0	0
	38	200	200	100	0	256	248

Positive patient sera were serially diluted for ELISAs (n = 2), microneutralization assay (n = 6), and PRNT_50_ (n = 2).

In the negative RBD IgG ELISA tier, there were five patients with neutralization antibodies (Table 2). These patients all had a high S IgG titer indicating there is an epitope other than the RBD which invokes the production of neutralizing antibodies^18^. Interestingly, three of these five patients had high S IgA titers, and these three patients had much higher neutralizing antibodies than the two patients with S IgG alone. In this tier, patients 30 and 34 with the highest neutralization titer also had RBD IgA.

In the low RBD IgG ELISA tier, patient 27 did not have neutralizing antibodies. This patient had RBD IgG levels barely above the cutoff and a modest amount of S IgG. The other 8 patient sera had varying degrees of neutralizing antibodies. Analyzing these 8 patients alone, a Pearson R value of 0.9198 was found when analyzing S IgG endpoint titers and microneutralization titers, indicating that when RBD IgG is not present there is a significant correlation between S IgG and neutralizing antibodies. Patients 21–25 demonstrated very high neutralizing titers. These patients all had significant levels of IgA antibodies. Of note, patient 23 had a significant level of S and RBD IgA and yielded the highest mean microneutralization titer (1:6502) of the low tier (Table 2).

The medium RBD IgG tier represented no correlation between S IgG endpoint and neutralization, whereas there was a slight positive correlation between RBD IgG endpoint and neutralization (Pearson r = 0.26). Patient 18 had the highest neutralization endpoint without the highest S or RBD IgG endpoints, again indicating specific S neutralization antibodies or neutralization due to S IgA. Patient 13 and 16 yielded the same S and RBD IgG endpoint titers however patient 16’s neutralizing endpoint was two-fold higher than patient 13 even though patient 13’s sera contained significantly more S and RBD IgA. This could be attributed to patient 16 having S neutralizing antibodies.

Patient 6 serum showed the most substantial neutralization activity in association with high S and RBD IgG endpoint titers, as well a considerable amount of IgA. In comparing patient 10 (bottom of high tier) to patient 11 (high of medium tier), their S and RBD IgG endpoint titers are identical yet patient 11 has a nearly twofold increase in neutralization endpoint. Again, the amount of S IgA from the serum of patient 11 is noticeably higher than patient 10.

### Healthcare worker analysis

From May 8, 2020 to May 21, 2020, we assessed the utility of our ELISA on serum samples donated by healthcare workers in Louisville, KY. The healthcare workers were initially screened for S IgG, S IgA, S IgM, RBD IgG, RBD IgA, and RBD IgM. Of the 1244 sufficient samples, 19 were confirmed positive for S IgG and/or RBD IgG (Table 3). Two patients (19 and 20) tested positive for active virus during the study. At the initial finger stick when active virus was confirmed via PCR, no antibodies were detected, however antibodies were detected on day 6 and 7 after viral diagnosis. It was determined that 13 of the 19 healthcare workers had neutralizing antibody titers ≥ 1:128, and therefore are potential convalescent plasma donors. In comparing the ELISA results to neutralization, the trend was the same as the 38 positive patients discussed above. Patients with low RBD IgG levels did not have neutralization antibodies unless there was a significant level of S antibodies present. This confirms the hypothesis of S containing a neutralizing epitope. When RBD IgG and S IgG levels are medium to high there is not a direct correlation with neutralizing endpoints, suggesting that differing S IgG antibodies and the potential that IgA antibodies may be neutralizing.

**Table 3:**
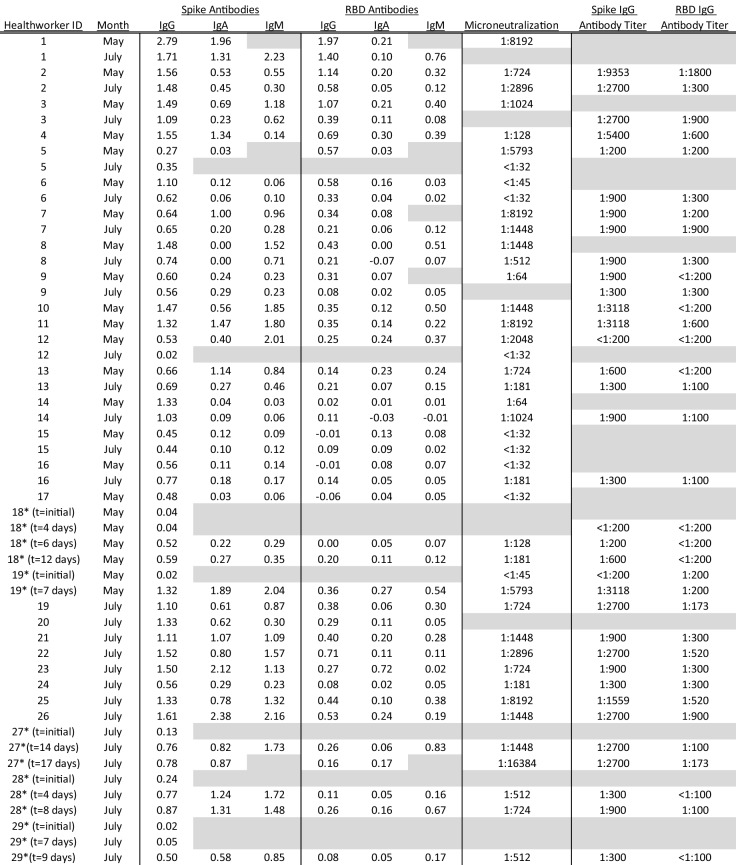
Screening of healthcare workers for SARS-CoV-2 antibodies.

Sera from healthcare workers was screened for S IgG, S IgA, S IgM, RBD IgG, RBD IgA, and RBD IgM antibodies by ELISA. Seropositive individual average values are shown for sera ran at a 1:100 dilution, n = 2. Positive sera were serially diluted for ELISAs (n = 2) and microneutralization assay (n = 2). Titers are expressed as geometric mean. Gray areas represent data not obtained due to insufficient sample.

**Figure 3:**
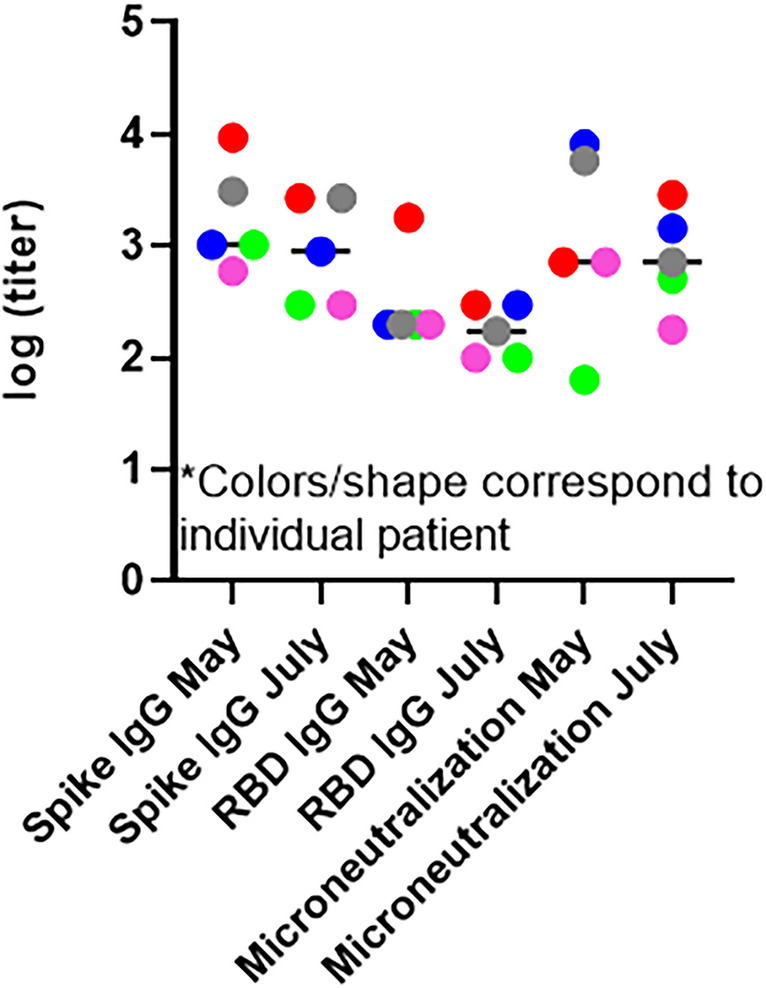
Comparison of round 1 and round 2 healthcare worker sera for SARS-CoV-2 antibodies. Log endpoint titers for seropositive healthcare workers (n = 5). Dot plot shows the comparison of Spike IgG, RBD IgG, and microneutralization from May to July. Colors correspond to individual healthcare worker.

In a second round of analysis of healthcare workers serum samples, collected between July 6–20, 2020, 932 individuals were tested for SARS-CoV-2 antibodies; 893 negative; 22 positive, 17 insufficient sample. The positivity rate is approximately 2.3%. Seventy four percent of the seropositive healthcare workers from Round 1 returned for antibody analysis. Of the 14 seropositive individuals who returned, 78.5% (11 of 14) maintained a high level of SARS-CoV-2 specific antibodies (Table 3). For 5 individuals, data for all end point titers was generated and compared (Fig. 3). There was no significant drop in overall titers, suggesting stability of antibody responses during the first couple of months of convalescence. During round 2, 3 individuals tested positive for active virus via RT-PCR. For all individuals with active infection, antibodies were not detected in the finger stick which was taken on the same day as the nasal swab. Antibodies were detected on day 4, 9, and 14 after PCR confirmed infection (Table 3).

## Discussion

Qualified serological assays are critical for epidemiological studies of SARS-CoV-2. Serological assays are necessary for not only understanding the nature of the immune response(s), but also allow a more accurate estimate of prevalence by including the rate of asymptomatic infections and more precise estimates of infection fatality rate, enhance contact tracing, aid evaluation of vaccine trials, and identify donors for the generation of convalescent serum/plasma therapeutics. Serology testing is debated due to the various test method performance characteristics, including choice of antigen, source of antigen, seroconversion time, and isotype switching. The highly immunogenic S protein, especially the RBD, is the target of neutralizing antibodies^8,19,20^. Recombinant S and RBD proteins have been produced in mammalian or insect cells, however mammalian cell produced antigens have superior performance in reactivity with SARS-CoV-2 sera^21^. N protein is known to be immunogenic, but N is highly homologous among coronaviruses, and therefore is expected to induce more cross-reactive antibodies. Long et al. found some COVID-19 patients with cross-reactivity to N of SARS-CoV but not S of SARS-CoV^6^. Seroconversion time must be considered when analyzing antibodies. Recent studies have revealed people rarely develop specific antibodies against SARS-CoV-2 within the first 7 days of symptoms. However, from day 7 to 14 IgM and IgG antibodies rapidly increase^6,22,23^. Therefore, to remove the timing limitation, we set our positive patient cohort for ELISA development, as RT-PCR plus antibody positive confirming all patients have seroconverted.

For our studies, we examined the same cohort of patients to understand the variation in serological responses to different SARS-CoV-2 antigens by studying IgG, IgM, and IgA to S, RBD, and N. All SARS-CoV-2 antigens were manufactured in mammalian cells and purified to a similar degree. ROC analysis revealed S and RBD having better sensitivity and specificity characteristics than N. Immunoglobulin to N gave the most varied response. IgG, IgA and IgM all demonstrated poor sensitivity and specificity and when combining IgG and IgM the combined positive predictive value (PPV) is only 29.2%, hence N is not the best antigen for SARS-CoV-2 diagnosis. In concordance with other studies, this suggests N has more cross-reactivity when compared with S and RBD and may not be the best antigen for clinical diagnosis.

The IgG, IgM or total antibodies are traditionally used for diagnosis having similar performance characteristics, however, a few studies have used SARS-CoV-2 specific IgA for diagnosis^1,24^. The detection of IgM antibodies may indicate a more recent infection and detection of IgG aids in examining the duration of immune response. Less is known about SARS-CoV-2 specific IgA, however, additional information gained about the IgA immune response is valuable for understanding SARS-CoV-2 infection. Overall, our analysis demonstrates that S may be the most robust detection antigen and that both—IgG and IgM are valuable immunoglobulins for diagnostic purposes. All SARS-CoV-2 infected volunteers developed IgG and IgM to S therefore no false negatives were found. However, IgG to S has a specificity of 96.6 leading to a few false positives. Analyzing IgG and IgM together yields a positive predictive value of 100%. Anti-S IgA gave a sensitivity of 94.7% therefore not all SARS-CoV-2 patients produce detectable IgA antibodies to S. IgG antibodies to RBD also gave a sensitivity of 94.7% showing some SARS-CoV-2 positive patients do not develop IgG to RBD at levels above our assay’s limit of detection.

In our study, the sensitivity and specificity of the RBD IgM and IgA ELISAs are not ideal, but this can be attributed to IgM being transiently produced in the early stages of infection only, and to the possibility that not all individuals produce detectable IgA. Other ELISA studies have shown differences in sensitivity and specificity between IgA and IgG based assays. A recent study found RBD IgA is more sensitive than RBD IgG 4–10 days after the onset of symptoms with the sensitivity of RBD IgG increasing 16 days after the onset of symptoms, thus the timing of blood sampling is important^24^. Thus, IgM ELISAs may be used as an additional supplemental analysis for RT-PCR to improve diagnosis of recent SARS-CoV-2 infection. IgA may be used to supplement diagnosis, and IgA response deserves further investigation.

The virus-neutralizing activity of serological samples confirm ELISA results due to neutralization specificity, identify immunoglobin levels need for “immunity”, and identify individuals for convalescent sera donation. Interestingly, we found patients that were negative or had low titers of RBD IgG but had high titers of S IgG, had neutralizing antibodies suggesting the possibility of another epitope besides RBD that produces neutralization. Furthermore, a significant correlation between S IgG and neutralizing antibodies was found in patients with low RBD titers. Taken together, these data indicate that S IgG antibodies exhibit strong neutralization capabilities against SARS-CoV-2. Although many studies have reported neutralizing antibodies against SARS-CoV-2 targeting the RBD of the S protein^25–29^, a recent publication in *Science* found neutralizing antibodies targeting an epitope on the N terminal domain (NTD) of the S protein that is independent of RBD in SARS-CoV-2^18^. Collectively, this supports our findings of high titers of S IgG exhibiting neutralization against SARS-CoV-2.

We have demonstrated differences in neutralization that are dependent on higher levels of S IgG and the possibility that S IgA antibodies are neutralizing when low titers of S IgG and S RBD occur. Recent serological studies have provided further insight into IgA and IgG antibody neutralization against SARS-CoV-2. IgG and IgA are known to play important roles in protection against respiratory viral infections^30–32^. IgG is the major antibody type produced systemically, while IgA is the most prevalent type on mucosal surfaces and provides protection against respiratory pathogens^30–32^. Previous studies have demonstrated that IgA is more effective than IgG in preventing influenza infections in both mice and humans^30,32–35^. Furthermore, IgA may have a role in SARS-CoV infections and immunity. Intranasal delivery of SARS-CoV proteins in mice provided better protection against viral replication in the lungs than intramuscular SARS-CoV challenge^30,36^. Additionally, intranasal MERS-derived vaccine reported neutralizing IgA antibodies in the bronchoalveolar lavage fluid in mice^30,37^. A more recent study evaluated the effectiveness of a chimpanzee adenoviral vaccine encoding stabilized spike protein from SARS-CoV-2 in mice^38^. This study compared intranasal and intramuscular delivery of the vaccine and concluded both routes of delivery resulted in protection from SARS-CoV-2 lung infection and pneumonia in mice^38^. Intranasal delivery induced a robust IgA and neutralizing antibody response which uniquely protected mice from both upper and lower respiratory tract SARS-CoV-2 infection and resulted in sterilizing immunity^38^. On the other hand, intramuscular administration did not induce an IgA response or result in sterilizing immunity^38^. Comparatively our study found examples of patients with neutralizing antibodies without extremely high titers of S or RBD IgG. This indicates that virus neutralization may be occurring due to higher titers of S IgA. For example, patient 11, who has much higher serum IgA, has nearly a two-fold increase in virus neutralization when compared to patient 10 (Table 2). Furthermore, a recent study found that SARS-CoV-2 infections resulted in early elevated titers of IgA neutralizing antibodies in patient serum, and a rapid decline in serum IgA neutralizing antibodies over time^30^. With only two patients studied we have seen this same trend (data not shown), further studies are needed to elucidate this observation.

Healthcare workers are a subpopulation thought to be at high risk for SARS-CoV-2 infection. In May of 2020, we enrolled healthcare workers from three Louisville, KY healthcare systems for concurrent viral load and antibody detection. The worker’s self-administered a nasal swab for RT-PCR analysis and a finger stick for serum collection. We identified 2 healthcare workers positive for active viral infection and 19 positive for S or RBD IgG antibodies for a seropositive rate of 1.4%. When this study was performed, the incidence rate in Kentucky was low (4.020 per 100,000 inhabitants), hence the 1.4% is not surprising. Similar to what we found, published studies have found various positivity rates in healthcare workers ranging from 1.6 to 31.6%^39–51^. Furthermore, comparisons to overall community infections rates are not included in all studies, but range from below the community spread to several times over community reported values^48–51^ which is consistent with the higher rate of infection seen in the healthcare workers when compared to the community.

Virus neutralization analysis of the healthcare workers in our study revealed that 13 of the 19 positive sera contained neutralizing antibodies. Some healthcare workers did not neutralize SAR-CoV-2 in microneutralization analysis. These individuals had IgG antibodies to SARS-CoV-2 S, and conversely did not have IgA or IgM nor any immunoglobulins to RBD. Two reasons for this are the presence of cross-reactive antibodies, especially for healthcare worker 14 or low levels of IgG to S. Healthcare worker 5 is an extremely rare find because in this individual the S IgG levels do not suggest seropositivity, however, there was significant RBD IgG and the antibodies neutralized the virus very well. Further studies are needed to confirm these findings. Two workers tested positive during the study and we were able to detect presence of S IgG and IgM 6 and 7 days after viral diagnosis. One patient had a very robust immune response showing a high level of all S immunoglobulins, RBD immunoglobulins and virus neutralization. A limitation of this phase of the study is the serum sample volume due to self-administered finger sticks as 128 workers did not have sufficient sample for testing and in some positive patients there was not enough sample to complete analysis endpoint titers or repeat analysis.

In the second round of our healthcare worker study we tested 932 individuals for SARS-CoV-2 antibodies. We found that the positivity rate increased from 1.4 to 2.3% in round 2, corresponding with the increased incident rate (11.847 per 100,000 inhabitants) in July for Kentucky. Notably 78.5% (11 of 14) of the healthcare workers that returned for round 2 maintained high levels of SARS-CoV-2 antibodies. For example, healthcare worker 2 maintained high levels of S and RBD IgG, IgA, and IgM along with virus neutralization from May to July. During round 2, we had 3 healthcare workers test positive for active virus by RT-PCR. Antibodies were not detected in the finger stick of these individuals, which was taken on the same day as the nasal swab, however the individuals developed detectable antibodies on day 4, 9, and 14 after viral diagnosis. In the future we will follow up with previously positive antibody or CPR healthcare workers and track their antibody development.

A limitation of our study is that most seropositive individuals we examined to validate ELISAs had a robust immune response (ELISA AUs greater than 1.0). In dissecting Fig. 1, for all scenarios, except N IgM, there is a significant difference between negative and positive antibody response. For example, the S IgG data (Fig. 1) shows there are only three patients between 0.2 and 1.0 AU. Consequently, for our ROC analysis there is a sharp distinction between sensitivity and specificity. However, analysis of the healthcare worker study reveals that positive patients have values between 0.2 and 1.0 AU. For example, patient 18, who was PCR positive in our study, had values of 0.52 and 0.59 AU on days 6 and 7 days after infection. This gray area (area between 0.2 and 1.0 AUs) shows some individuals with neutralizing responses and some without, therefore an increase in the SARS-CoV-2 positive cohort will provide improvement to ROC analysis. In addition, it is useful to look at IgM and IgA levels to determine seropositive patients and predict neutralization activity. To determine the root cause of this gray area, further analysis is ongoing in our laboratory as we analyze more seropositive individuals. It is likely that disease severity and timing post infection impact the diagnostic performance. A recent study has shown a clear distinction between symptomatic and asymptomatic individuals, with symptomatic individuals having a statistically higher RBD IgG titer. Furthermore, the study found diagnostic sensitivity and specificity values are different when considering symptomatic and asymptomatic patients separately as well as the timing of when antibodies are measured after infection^52^.

Qualified serological assays will continue to be an important tool and are central to disclosing accurate information during the SARS-CoV-2 pandemic. The sample size for our positive and negative cohort are average, hence as we continue to collect patient samples we intend to update our ROC analysis for continuous improvement of ELISA threshold values and performance characteristics. A serological toolbox to measure and understand antibody responses to SARS-CoV-2 are continuously vital to understanding the current pandemic and monitoring immunity for vaccine development. This study provides valuable data on ELISA based serological testing, correlations with neutralization, understanding the broad immune response to SARS-CoV-2 and seropositive rates in healthcare workers.

## Methods

### Human samples for qualification

Serum samples were obtained from Norton Healthcare, Louisville, KY. Subjects with confirmed SARS-COV-2 infection via RT-PCR were eligible to donate serum per institutional protocol (IRB 20.0380). Samples of these subjects were confirmed by serology for IgM or IgG utilizing Premier Biotech COVID-19 IgG/IgM Rapid Test Cassette. For ELISA analysis samples were de-identified and tested with our developed method.

Pre-COVID-19 samples were obtained from samples collected prior to October 2019 per institution protocol 11.0432 and 12.0274.

### RT-PCR method

The detection of viral RNA was performed as described by the FDA (https://www.fda.gov/media/134922/download), with minor revisions. Nasal or nasopharyngeal swabs were diluted in Trizol, and RNA was extracted using the Direct-zol 96 MagBead RNA kit following the manufacturer’s instructions. The washing process was automated using a Freedom Evo (Tecan) liquid handler and MultiFlo (Biotek) dispenser. The quantitative polymerase chain reaction master mix was made using TaqPath 1-Step RT-qPCR Master Mix, CG and the 2019-nCoV CDC qPCR Probe Assay. The PCR reaction was performed using a QuantStudio 7 Pro, and samples were analyzed with Design and Analysis Software version 2.4.

### ELISA method

Serum samples were analyzed for IgG, IgA, and IgM antibodies against SARS-CoV-2 S, RBD, and N antigens. MaxiSorp 96-well plates were coated with respective antigen (Spike Protein from Novel Coronavirus (SARS-CoV-2/Wuhan/2019) Trimeric, Immune Technology Corp, # IT-002-032p; Spike protein RBD from Coronavirus SARS-CoV-2 (CoVID-19/Wuhan), His-tagged, Immune Technology Corp, # IT-002-036p; Nucleocapsid Protein from Coronavirus SARS-CoV-2 (CoVID-19/Wuhan), Immune Technology Corp, # IT-002-033Ep) at 2.5 µg/ml in PBS buffer overnight at 4 °C. Plates were blocked with protein free buffer (ThermoFisher Scientific). Serum samples were diluted to 1:100 in protein free buffer and 100 µl of sample/well were placed on the plate in duplicates and incubated for 30 min at 37 °C. For detection, anti-human IgG-HRP (Rabbit anti-human IgG-HRP, Thermo Fisher #PA128587), anti-human IgA-HRP (Goat Anti-Human IgA, HRP, Southern Biotech 2050-05), or anti-human IgM-HRP (Goat Anti-Human IgM, HRP, Southern Biotech 2020-05) antibodies were added to the wells, and incubated for 1 h at room temperature. The plates were washed three times with PBST between each of these steps. Plates were developed using a tetramethylbenzidine substrate and ELISA stop solution (1 N HCl) and read at 450 nm. Receiver-operating characteristic (ROC) analysis was used to determine the positive threshold value for each ELISA (Table 1). For analysis with serial dilutions of samples the ELISA method was the same with the 1:100 dilution of sera sample diluted threefold down the plate in protein free buffer. All values were blank subtracted, and the endpoint titer was determined with a cutoff value of 0.1.

### Microneutralization method

Vero E6 cells were seeded at a density of 2 × 10^4^ cells in 96 well tissue culture plates (Corning/Costar) and incubated overnight at 37 °C with 5% CO_2_. The following morning, cells were washed once with 100 µl DMEM containing penicillin/streptomycin and 5% FBS (VIM) followed by two washes with 200 µl of PBS prior to the addition of 100 µl of VIM. Prior to the addition of virus, twofold serial dilutions of human serum samples were made in VIM in a separate 96 well dilution plate. SARS-CoV-2 was then added to the dilution plate at a concentration of 60 pfu/well for a final MOI of 0.05 and incubated with the serum for 1 h at 37 °C, 5% CO_2_. Following this incubation, media on the cells was replaced with 100 µl of the serum and virus mixture and returned to the incubator for four days. At the conclusion of incubation, cells were fixed with 4% paraformaldehyde and stained with 1% crystal violet for 20 min at room temperature. Cells were then washed twice with 200 µl of filtered tap water and assessed for CPE.

### PRNT method

Vero cells were seeded at 1 × 10^5^ cells/well in 24-well tissue culture plates (Corning/Costar) and incubated overnight at 37 °C with 5% CO_2_ until approximately 95% confluency. SARS-CoV-2 human serum samples obtained were diluted at 1:32 followed by twofold serial dilutions. For samples with high neutralization activity, the PRNT was retested with increased serum dilutions by fourfold serial dilution at 1:64 up to 1: 31,250 dilution. SARS-CoV-2 (USA-WA-1, 3 × 10^6^ PFU) was diluted in DMEM, 10% FBS, 25 mM HEPES to yield 30–40 plaques/well (0.5 MOI) in the virus control wells. An equal volume of SARS-CoV-2 was added to each diluted serum sample, and the virus-serum mixture was incubated at 37 °C with 5% CO_2_ for 60 min. The cell culture medium was aspirated, and the virus-serum mixture (150 µl/well) was transferred onto 24-well plates and incubated at 37 °C with 5% CO_2_ for 60 min. The virus-serum inoculum was aspirated and 0.5 ml of Avicel overlay medium (1%, FMC chemical) in EMEM supplemented with 5% heat-inactivated FBS, 25 mM HEPES, 0.2% Sodium Bicarbonate, and 1% antibiotic/antimycotic solution was added to each well. Incubate at 37 °C, 5% CO_2_ for three days. The overlay medium was removed, and the cell monolayers were washed twice with 1 × PBS. The cells were fixed and stained with Crystal violet/paraformaldehyde solution. The plates were rinsed with tap water and allowed to air dry. Plaques were counted from each well, and the standard curves were drawn by calculating the plaque numbers at different dilution rates that were transformed by the log_2_ or log_4_ values. The maximum plaque numbers (30–40) obtained from the control plate at each experiment were used to find 50% plaque formation, and the PRNT_50_ was calculated from the standard curves.

### Healthcare worker study design and sampling

Healthcare workers who had been actively working in areas with patient contact after mid-March in Louisville, KY healthcare systems were invited to participate in a study to look at infection rates and immune responses to SARS-CoV-2 (University of Louisville IRB# 20.0312). Recruitment was through routine health system communications such as newsletters and weekly COVID-19 update phone calls. Participants need to have worked outside their home during the 30 days prior to the start of the study in mid-May 2020. In May, at centralized locations, approximately 1300 participants picked up kits with sampling instructions and self-collected nasal swab samples with polyester flock tipped swabs and serum via finger stick in microtainers (BD 365,967). Samples were returned within an hour of collection and refrigerated until processing. For processing, samples were centrifuged at 5000×*g* for 5 min, serum was collected, and stored at − 20 °C until use. Not all participants were able to self-collect a serum sample, we received 128 insufficient samples from approximately 1300 participants. In July, at centralized locations, approximately 1000 participants picked up kits and completed the visit as described above. Not all participants were able to self-collect a serum sample, we received 17 insufficient samples from 1000 participants. All method and experimental protocols were carried out in accordance to relevant guidelines and regulations, approved by institutional protocols, and informed consent was obtained.
